# Insights Into Urogenital Tuberculosis: Impact of Microbiological Diagnosis

**DOI:** 10.7759/cureus.113963

**Published:** 2026-08-04

**Authors:** Kiran Bala, Rojaleen Das, Neeta Singla, Amlesh Seth, Urvashi B Singh

**Affiliations:** 1 Microbiology, All India Institute of Medical Sciences, New Delhi, New Delhi, IND; 2 Epidemiology and Public Health, National Institute of Tuberculosis and Respiratory Diseases, New Delhi, IND; 3 Urology, All India Institute of Medical Sciences, New Delhi, New Delhi, IND

**Keywords:** epididymo-orchitis, genital tuberculosis, mycobacterium tuberculosis, rifampicin resistant tb, urogenital tuberculosis

## Abstract

Many varied presentations of genitourinary tuberculosis (GUTB) such as recurrent urinary tract infection, sterile pyuria (pus cells in urine despite a negative routine bacterial culture) with/without haematuria, irritating voiding symptoms, infertility, hydronephrosis and renal failure had been published in the literature. Tuberculous epididymo-orchitis (inflammation of the epididymis and testis) is an underreported and rare form of GUTB. Non-specific symptoms of epididymal tuberculosis (TB) are difficult to differentiate from cysts, tumours and other infections. Bilateral epididymal involvement with testicular lesion increases the suspicion of tubercular aetiology. Tuberculous epididymo-orchitis in late stages may present with non-functioning kidney and thimble bladder (a severely contracted, small-capacity bladder due to chronic inflammation) and infertility. Timely diagnosis and treatment will prevent late sequelae of the disease. Here, we present three cases of GUTB in young males: two cases of isolated tuberculous epididymo-orchitis (cases 1 and 3) and one case of complicated drug-resistant genitourinary tuberculosis (case 2). These rare cases were managed successfully with diagnostic and therapeutic challenges.

## Introduction

Genitourinary tuberculosis (GUTB) refers to tuberculosis of the female and male genital tract and the urinary tract. Male genital tuberculosis is usually associated with renal tuberculosis in 60% to 65% of cases and with pulmonary tuberculosis in 34% of cases [1]. GUTB accounts for approximately 20%-40% of extrapulmonary tuberculosis (TB) cases in endemic regions. The disease usually results from haematogenous dissemination of *Mycobacterium tuberculosis* to the kidneys, with subsequent spread to other parts of the genitourinary tract. Epididymis is the most common site for male genital TB, and direct extension may lead to testicular involvement and extra-testicular extension to the scrotum [1,2]. GUTB may manifest firstly as vague non-specific lower urinary tract symptoms such as dysuria, urgency and frequency, diverse semen or urine biochemical and pathological analysis and “sterile” pyuria (pus cells in urine despite a negative routine bacterial culture) and/or haematuria [3]. Most of times patients had received multiple courses of empirical broad-spectrum antibiotics with or without evidence of bacterial infection or despite a sterile pyuria report. Diagnostic challenges always persist due to non-specific symptoms, mimicking recurrent bacterial UTI and inconclusive radiological, microbiological and histopathological investigations. It leads to non-resolution of symptoms and continuing spread of tubercular disease to adjacent organs with further deterioration of symptoms and complications related to the renal and genital system [2,3]. Samples (pus discharge, biopsy, semen, prostatic secretions, urine) should be subjected to complete panel of microbiological investigations such as Ziehl-Neelsen (ZN) smear, molecular testing (GeneXpert (Cepheid, Sunnyvale, California, United States), TB-PCR) and culture (Lowenstein Jensen (LJ) culture LJ, Mycobacterial Growth Indicator Tube (MGIT) (Becton, Dickinson and Company (BD), New Jersey, United States) culture). Our three cases of GUTB were diagnosed based on a combination of clinical presentation, radiological findings, histopathology and microbiological confirmation modalities (ZN stain and culture: case 1, GeneXpert: case 2 and MGIT and LJ culture: case 3). Representative sample was pus sample in two isolated cases of tuberculous epididymo-orchitis and semen sample in genitourinary tuberculosis (case 2). Pulmonary involvement (past or present) was not seen in all cases. The prompt and accurate diagnosis can guide appropriate and efficient treatment. We are presenting two cases of genital TB and one case of complicated drug-resistant urogenital TB in young immunocompetent males. This case series describes the varied clinical presentations of genitourinary tuberculosis and highlights the diagnostic challenges associated with atypical manifestations, aiming to improve clinical recognition and facilitate timely diagnosis. This article was previously posted to the microbiology research preprint server on July 24, 2023.

## Case presentation

Case 1

A 20-year-old male from Manipur in the north-eastern part of India presented to the Surgical Out-Patient Department with chief complaints of pain and swelling in the left scrotum for the last three weeks. He gave a history of scrotal pain for the past one week, after which he noticed a swelling on the left side of the scrotum. Initially, he was treated with antibiotics and anti-inflammatory drugs before presenting to our centre, but there was no relief from the symptoms. The patient was afebrile and had no complaints of dysuria, haematuria or frequent voiding. There was no episode of back pain, flank pain or abdominal pain. There was no past history of TB, diabetes or any other chronic illness, or history of contact with TB. On local examination, an enlarged left side of the scrotum with thickened left epididymis was observed.

**Figure 1:**
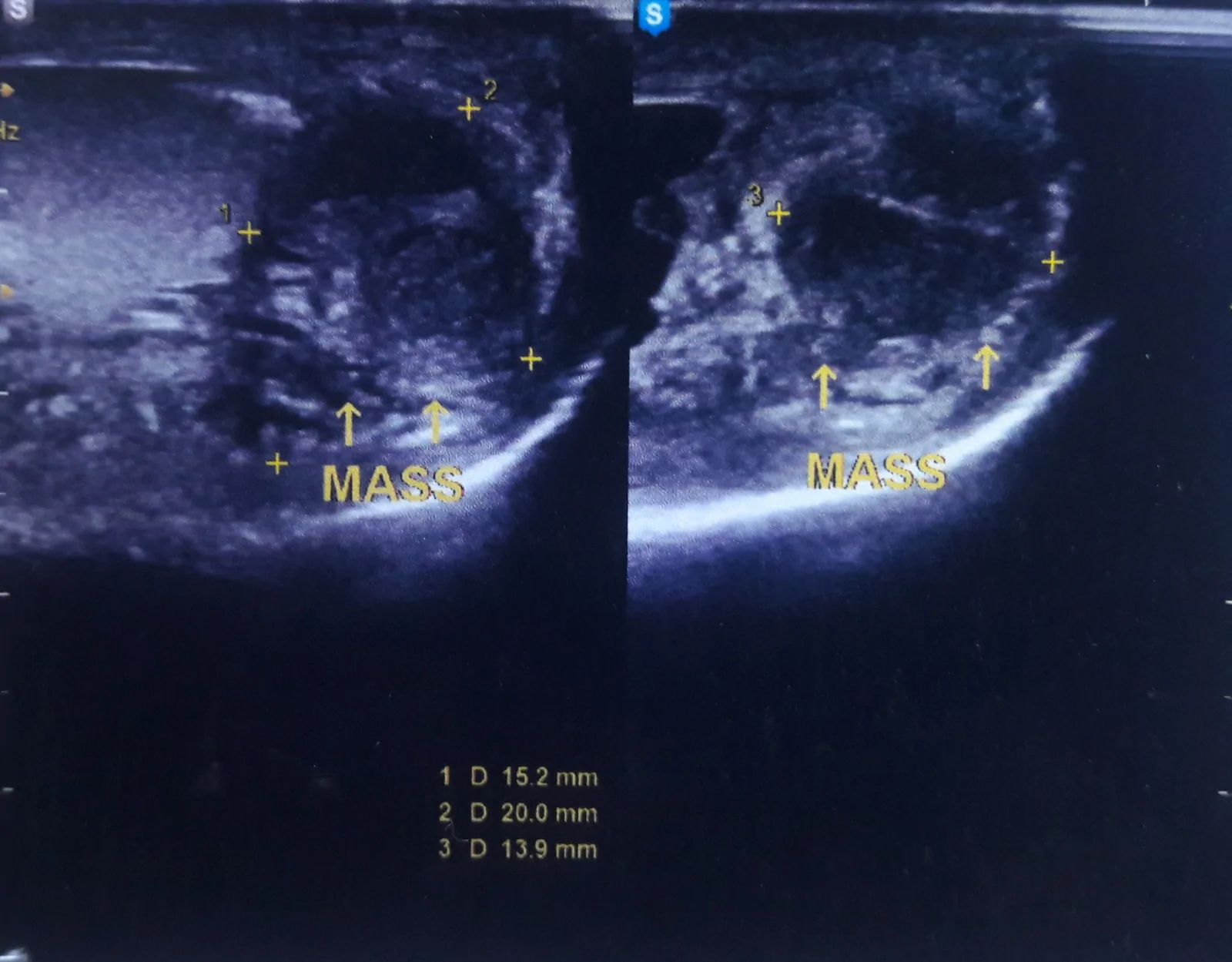
Scrotal ultrasound showing mass near inferior part of left epididymis of case 1. Arrows are directing towards mass (significant lesion) and plus sign is measuring the area of mass.

**Figure 2:**
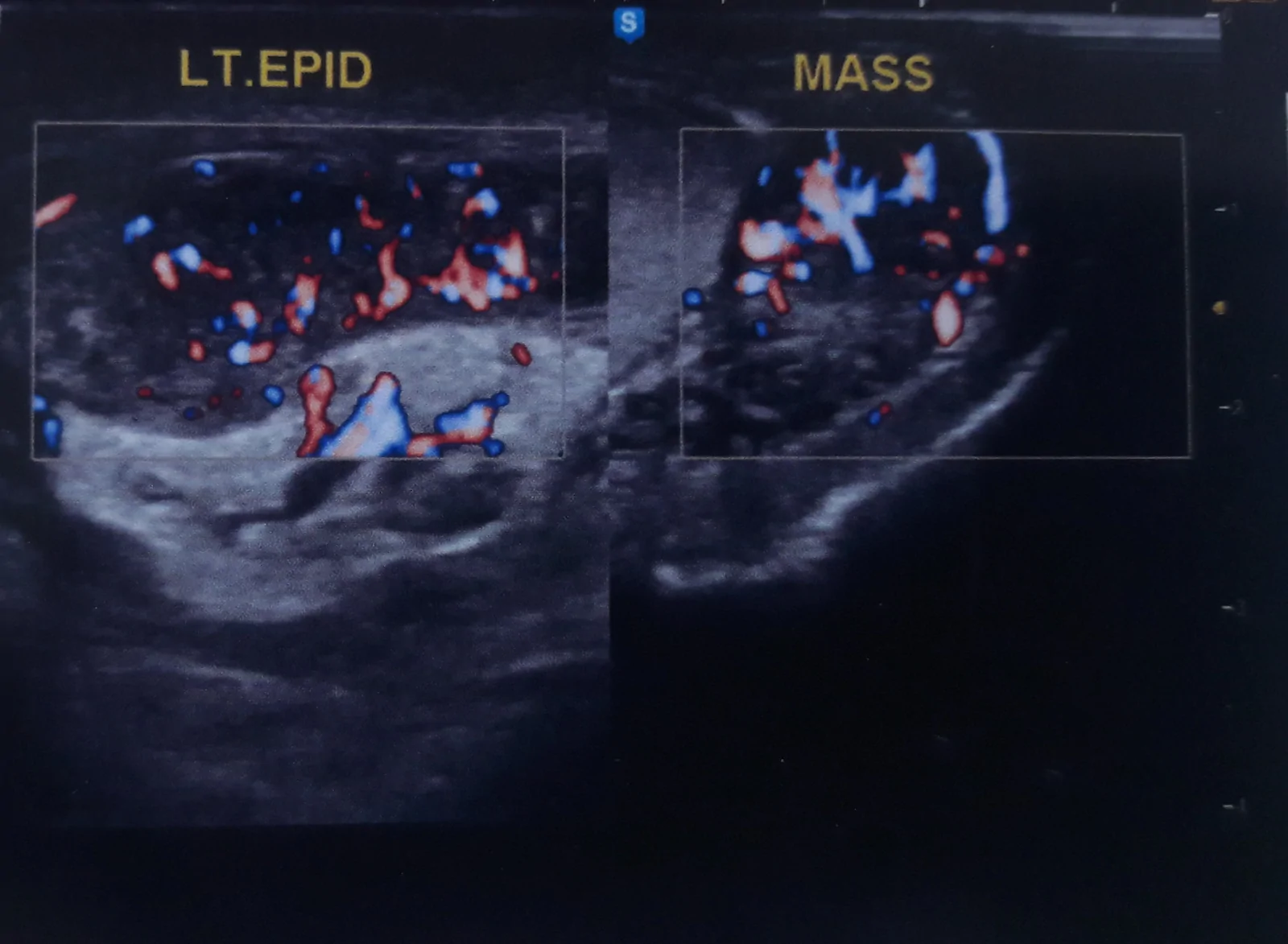
Scrotal Doppler showing increased vascularity in left epididymis of case 1. LT = left; EPID = epididymis.

Radiological investigations such as scrotal ultrasonography (Figure 1) and scrotal Doppler (Figure 2) were performed. Tuberculosis was suspected on scrotal ultrasonography (Table 1) and scrotal Doppler (Figure 2). Fine needle aspiration cytology (FNAC) was performed, and pus sample was subjected to microbiological investigations, and GeneXpert detected *Mycobacterium tuberculosis*, which is sensitive to Rifampicin. MGIT culture showed growth after 3 weeks which was confirmed as *M. tuberculosis*.

**Table 1:** Investigations of case 1. FNAC = fine needle aspiration cytology; ZN = Ziehl-Neelsen; MGIT = Mycobacterial Growth Indicator Tube; AFB = acid-fast bacilli; M.Tb/Rif = *Mycobacterium tuberculosis*/Rifampicin; TBCID = Tuberculosis Culture Identification.

Investigations of Case 1		
Radiological Investigations	Scrotal ultrasonography (Figure 1)	Thickening of the head, neck and tail of the left epididymis. Heteroechoic inflammatory mass was seen near the inferior pole of the left epididymis measuring 20×15×13 mm. Two small hypoechoic areas within thickened tail of epididymis measuring (7×6 mm and 6×4 mm) suggestive of micro-abscesses.
	Scrotal Doppler (Figure 2)	Increased vascularity in left epididymis.
	Chest X-ray	No primary focus found.
Microbiological Investigations	Pus aspirate (FNAC)	
	ZN staining	Positive for acid-fast bacilli.
	GeneXpert M.Tb/Rif	*M. tuberculosis* detected; Rifampicin sensitive.
	MGIT automated liquid culture	Positive after 3 weeks; confirmed as *M. tuberculosi*s by biochemical test and TBCID.
	ZN staining (sputum three consecutive samples)	Negative for AFB.

Diagnostic red flags for case 1 are persistent scrotal swelling and pain despite antibiotic therapy, thickened epididymis, and imaging findings suggestive of tuberculosis should raise suspicion of GUTB. Microbiological confirmation from aspirated pus sample is essential for accurate diagnosis. The patient was started on anti-tubercular therapy (ATT) with intensive phase drugs consisting of Rifampicin, Isoniazid, Ethambutol and Pyrazinamide daily for two months, followed by four-month course of continuation phase Rifampicin, Isoniazid and Ethambutol. The swelling of the scrotum reduced and pain was relieved after two weeks, and the patient was asymptomatic after completion of therapy; radiological findings were normal, and microbiological analysis was negative for *M. tuberculosis*.

Case 2

A 29-year-old unmarried male presented to the surgical Outpatient Department (OPD) in February 2014 with complaints of pain in left flank region and swelling in the left testicle for last six months. He has been complaining of burning micturition for the last two months. The initial urinary symptoms were suggestive of a chronic lower urinary tract infection. There was history of loss of appetite and significant weight loss. He had no past history of tuberculosis, diabetes or hypertension or any history of contact with TB. His physical examination appeared normal (Appendix 1).

**Figure 3:**
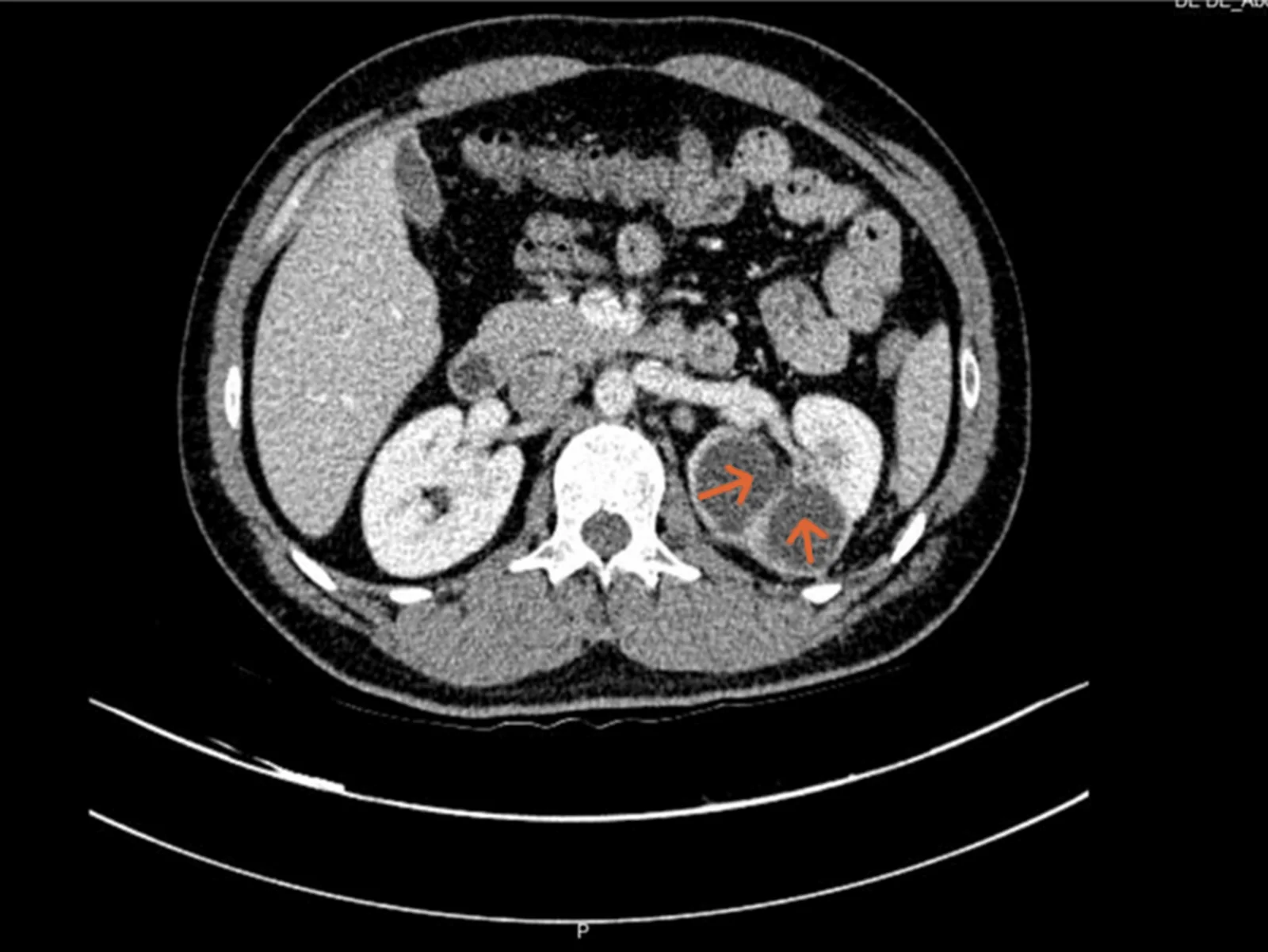
CT urogram showing calyceal neck stricture in left kidney (arrows) of case 2.

Ultrasound of the abdomen and pelvis and fine needle aspiration cytology (FNAC) of the epididymis was performed which gave inconclusive results (results discussed in Table 2). Microbiological investigations (ZN stain, GeneXpert) of FNAC sample and urine sample were negative. The patient was started on ATT based on histopathological evidence of granuloma. At this stage, microbiological confirmation and drug susceptibility testing could not establish the diagnosis (ZN stain and GeneXpert urine and FNAC sample were negative), and treatment was initiated based on clinical and histopathological findings. The patient did not show any sign of improvement after two months of intensive phase of ATT, and his condition further deteriorated with urinary obstruction. Procedure of left double J stent (DJ) stenting was performed to relieve the symptoms of urinary obstruction. Meanwhile ATT was continued, and stent was removed after three months. After completion of five months of ATT, patient’s symptoms worsened with increase in left flank pain along with testicular swelling. He had to undergo open drainage and nephrostomy for symptomatic relief. Biopsy sample of epididymis was taken and subjected to histopathology which revealed granulomatous epididymitis (Table 2). Due to persistent symptoms despite prolonged ATT, further evaluation was performed to confirm the diagnosis and assess possible drug resistance. After three months following last intervention due to non-resolution of symptoms, he was advised contrast-enhanced computed tomography (CECT) abdomen (Figure 3) and complete microbiological analysis (microscopy, culture, molecular) of semen sample and urine (over five consecutive days) (Table 2). Molecular testing was performed on the semen sample because of the predominant genital involvement, including persistent epididymal and testicular symptoms. GeneXpert performed on the semen sample detected rifampicin-resistant *M. tuberculosis*, suggestive of multidrug-resistant (MDR) TB.

**Table 2:** Investigations of case 2. HPF = high-power field; RBC = red blood cell; HIV = human immunodeficiency virus; HBsAg = hepatitis B surface antigen; HCV = hepatitis C virus; KFT = kidney function test; LFT = liver function test; CT = computed tomography; FNAC = fine needle aspiration cytology; ZN = Ziehl-Neelsen; MGIT = Mycobacterial Growth Indicator Tube; TB PCR = tuberculosis polymerase chain reaction; MDR = multidrug resistant; ATT = anti-tubercular treatment; M.Tb/Rif = *Mycobacterium tuberculosis*/Rifampicin.

Investigations of Case 2		
Routine Investigations	Urine analysis	pH:6.0, Leucocyte count: 2-4/HPF, RBC: 0-1/HPF, no crystals or cast seen. (Biological reference value, pH: 4-8, RBC: 0-3/HPF, leucocyte count: 0-2/HPF).
	Blood investigations	HIV: Negative. HBsAg: Negative. HCV: Negative. KFT/LFT: within normal limits.
Radiological Investigations	Abdomen and pelvis ultrasound	Right kidney measures 11.3 cm; Left Kidney: 10.4 cm. 5 cm×3 cm cystic lesion on the upper pole of left kidney. Diagnosed as differential hydronephrosis.
	CT urogram (Figure 3)	Left superior hydrocalyx causing cortical atrophy secondary to calyceal neck stricture along with short segment areas of non-opacification in left proximal (2.5 cm) and left distal ureter (3.3 cm) with its upwards dilatation suggestive of ureteric formation.
Histopathology	Histopathology of Aspirate	Necrotizing granulomatous epididymis
	Histopathology of epididymal tissue	Granulomatous epididymitis
Microbiological Investigations	Urine culture	Negative
	ZN staining, GeneXpert of Aspirate (FNAC) at time of presentation, after 5 months	Negative
	ZN staining, GeneXpert on consecutive 5 pooled urine samples at time of presentation, after 5 months	Negative
	ZN staining of semen sample at 5 month	Negative
	Urine and semen culture on LJ media 5 month	Negative
	GeneXpert M.Tb/Rif on semen sample at 5 month	*M. tuberculosis* detected; Rifampicin resistant.
Post ATT		
Radiological Investigations	Abdomen and pelvis ultrasound	Ill-defined lesion of size 4.7x 2.9 cm along with parenchymal thinning.
Microbiological Investigations	ZN staining of semen	Negative
	MGIT (semen)	Negative
	TB PCR (semen)	Negative
	GeneXpert (semen)	Negative

The patient was now initiated on ATT against multidrug-resistant (MDR) TB on the basis of GeneXpert report of semen sample. DJ stenting and nephrostomy intervention was done again. After six months of treatment, his symptoms were relieved and his general well-being had improved. Earlier microbiological evaluation, including molecular testing and drug susceptibility assessment of semen sample, may have facilitated earlier detection of MDR-TB and potentially prevented prolonged ineffective therapy and repeated interventions. He was evaluated for cystic swelling of the kidney through ultrasonography, and semen was subjected to investigations (Table 2) after five months of ATT, and it was negative for *M. tuberculosis*. Diagnostic red flags for case 2 are persistent flank pain with epididymal swelling, urinary obstruction, worsening symptoms despite adequate ATT, and recurrent disease progression indicating possible drug-resistant genitourinary tuberculosis (drug-resistant GUTB).

Case 3

A 35-year-old male presented to surgery emergency with complaints of right side scrotal swelling, pain, dysuria associated with pus discharge since one month. He has complaint of acute onset of right scrotal swelling with dysuria one month back and consulted a physician and it was diagnosed as bacterial urinary tract infection and broad spectrum antibiotics was prescribed. But there was no relief in the symptoms after 15 days of medications. Patient condition deteriorated and presented to emergency. He was admitted and testicular torsion was suspected, and ultrasonography (USG) of scrotum was advised. Testicular torsion was ruled out on the basis of USG report and urology consultation. Broad spectrum antibiotics were prescribed again but patient again reported with increase in pain, pus discharge after three days. There is past history of similar episode of sudden onset left side scrotal swelling associated with pus discharge and evening rise of fever which was relieved after taking broad spectrum antibiotics 1.5 years back. He was doing well after this episode. There was no episode of back pain, flank pain or abdominal pain. There was no past history of TB, diabetes or any other chronic illness, or history of contact with TB. The persistence of symptoms despite prolonged therapy, associated systemic features, and granulomatous inflammation prompted further evaluation for GUTB. Urogenital tuberculosis was suspected on scrotal ultrasonography, and aspirated pus sample was subjected to microbiological investigations and *M. tuberculosis* was detected on culture (Table 3).

**Table 3:** Investigations of case 3. FNAC = fine needle aspiration cytology; ZN = Ziehl-Neelsen; M.Tb/Rif = *Mycobacterium tuberculosis*/Rifampicin; TBCID = Tuberculosis Culture Identification.

Investigations of Case 3		
Radiological Investigations	Scrotal ultrasonography	Scrotal ultrasonography revealed thickening of head, neck and tail of right epididymis. Heteroechoic inflammatory mass was seen near inferior pole of right epididymis measuring 18×12×13 mm.
	Chest X-ray	No primary focus found.
Microbiological Investigations	Pus aspirate (FNAC)	
	ZN staining	Negative for acid-fast bacilli (AFB).
	GeneXpert M.Tb/Rif (Cepheid, CA, USA)	*M. tuberculosis* not detected.
	Lowenstein Jensen culture	Positive after 3 weeks; confirmed as *M. tuberculosis* by biochemical test.
	Mycobacterial Growth Indicator Tube (MGIT 960 liquid culture; Becton Dickinson, Sparks, MD, USA)	Positive after 3 weeks; confirmed as *M. tuberculosis* by biochemical test and TBCID.
	ZN staining (Sputum three consecutive samples)	Negative for AFB.
Routine Investigations	Blood investigations	Liver function tests/kidney function tests are within the normal limits.

Diagnostic red flags for case 3 are recurrent scrotal swelling with pus discharge, poor response to broad-spectrum antibiotics, and relapse of symptoms after previous episodes should raise suspicion of genitourinary tuberculosis. Confirmation through imaging and microbiological testing is important for accurate diagnosis. The patient was started on ATT with intensive phase drugs for two months, followed by four-month course of continuation phase Rifampicin, Isoniazid and Ethambutol. The swelling of the scrotum reduced, and pain was relieved with no pus discharge after 12 days, and the patient was asymptomatic, and radiological examination was normal and microbiological analysis was negative for *M. tuberculosis* after completion of therapy. The patients of cases 1, 2 and 3 were followed for one and half years and all were healthy without any complications.

## Discussion

GUTB is the most common presentation of extrapulmonary tuberculosis after lymphatic involvement [4]. GUTB most commonly affects the kidneys, followed by the ureter and urinary bladder. In case of males, the prostate, seminal vesicles, deferent ducts, epididymis, Cooper's glands, penis, and testicles may be affected [4]. Most of the time, silent bacillaemia occurs due to associated pulmonary tuberculosis. However, active lesions in the kidney and genital organs may remain clinically dormant for even years together. When this infection gets reactivated, it may cause destruction of the renal parenchyma and further leads to strictures, obstruction, and loss of renal function [5,6]. Genital TB is often associated with renal TB, but it may also present in isolation [1,3,7-9]. It is found that primary sites of infection are the epididymis in men and fallopian tubes in females [7,8,9]. Similar to previous reports, our patients presented with epididymal swelling and delayed diagnosis. In this case series, the first and third cases presented with isolated tubercular epididymo-orchitis in young males with no past history of tuberculosis and no evidence of pulmonary tuberculosis presently. Krishnamoorthy et al. published that 22.7% had associated past history of treated pulmonary tuberculosis with a mean duration of 3-10 years [3].

Most commonly, the epididymis is involved in 10% to 55% of males with GUTB [3,8]. A few case reports of isolated epididymo-orchitis have been published in the literature. Cherif et al. reported an isolated case of epididymo-orchitis in AIDS patients, and from India, Shenoy et al. have reported a case of bilateral epididymitis with right-sided orchitis [8,9,10-15]. Sharma et al. highlighted the diagnostic efficiency of FNAC in tubercular epididymo-orchitis [16]. All of these patients presented with pain and scrotal swelling and pus discharge with sterile pyuria and intake of multiple courses of antibiotics in due course of time [8-16]. Whenever it is associated with renal pathology, loin pain is most common symptom followed by burning micturition, dysuria and haematuria [1,3]. Diagnostic modalities such as radiological investigations such as CT scan, MRI, USG scrotum and histopathology and microbiological investigations supported in confirmation of diagnosis and efficient treatment [3,17].

Radiological imaging is up to 91.4% sensitive for diagnosis of GUTB and has a cardinal role in localizing the foci of the disease, the damage extension and applied for targeting the biopsies [18]. Representative sample from site of disease pathology include urine samples (three consecutive early-morning, first-void midstream) semen, tissue specimens, pus, or discharged or prostatic massage fluid. Microbiologically confirmed diagnosis could be obtained using microscopic examination using Ziehl-Neelsen (ZN) or auramine staining, culture (LJ, Mycobacterial Growth Indicator Tube (MGIT)), molecular testing (GeneXpert, Truenat (Molbio Diagnostics Limited, Goa, India), PCR). AFB smear microscopy has approximated 20%-23% sensitivity, culture has long turnaround time (2-8 weeks) with 5%-10% sensitivity and molecular tests have 30%-38% sensitivity [1,3,18,19]. Case 3 was microbiologically confirmed by LJ culture, which is infrequently reported in isolated epididymal TB.

The second case was MDR-GUTB involving kidneys as well as male genitalia. The radiological changes in the patient’s kidney suggested long-standing disease, which may also possibly explain the genesis of MDR-TB. Kidney fibrosis extended to the urinary collecting system and lead to hydronephrosis that required stenting [18]. Few other authors have reported MDR-GUTB [19,20]. Unlike most reported cases, case 2 developed rifampicin-resistant (MDR) genitourinary TB despite initial empirical ATT. It becomes essential to diagnose MDR-TB as the patient has undergone courses of anti-tubercular treatment without any improvement and correct diagnosis could not be made despite best efforts and using all diagnostic modalities for urine and pus sample. Furthermore, GeneXpert of semen sample yielded the accurate diagnosis of MDR-TB and precise treatment could be provided.

Due to varied non-specific symptoms and insidious and chronic presentation of GUTB with delayed diagnosis, leading to a high rate of urogenital organ impairment, more than 50% of patients require surgical management with anti-tubercular therapy for drug-sensitive and drug-resistant tuberculosis [1,3,18]. Whenever renal system is impaired with manifestations of non-visualised kidney in (23%), hydronephrosis or hydroureteronephrosis (31%) and distortion, cavitation or scarring of the calyces (14.5%), thimble bladder (6.2%), surgical procedures such as nephrectomy and reconstructive procedures, percutaneous nephrostomy and internal DJ stenting are recommended along with ATT [3]. GUTB patients may relapse at a higher rate than pulmonary TB, in 6.3% to 22% of cases, even after 12 months of medical therapy [19,20]. Our three patients presented with epididymal involvement; they differed in microbiological findings, disease progression, and treatment response. Case 2 demonstrated rifampicin-resistant tuberculosis with delayed diagnosis and showed clinical improvement after MDR-TB treatment with negative follow-up microbiological testing, whereas cases 1 and 3 responded to first-line ATT following microbiological confirmation and remained asymptomatic after completion of ATT. Follow-up should be done for these patients. Two cases were managed with ATT, and second case had to undergone invasive procedures, DJ stenting and nephrostomy along with multidrug-resistant regimen of ATT. These cases highlight key clinical lessons as case 1 demonstrates the value of early molecular testing for rapid diagnosis and timely initiation of appropriate therapy. Case 2 emphasizes the need for early drug resistance testing in patients with persistent symptoms despite anti-tubercular therapy to avoid delays in diagnosing MDR-TB. Case 3 underscores the importance of combining molecular assays with culture to improve the diagnostic accuracy of extrapulmonary tuberculosis. Limitations of this case series is limited by its small sample size and single-centre design, which may limit the generalizability of the findings. Larger studies are needed to validate these observations.

## Conclusions

GUTB should be suspected in patients with a history of multiple episodes of antibiotic therapy due to recurrent urinary tract infection with sterile pyuria. Our findings suggest we could reach an accurate diagnosis with a panel of all kinds of representative samples and using all diagnostic modalities such as microbiological, radiological and histopathological. Microbiological detection of rifampicin-resistant tuberculosis helped in the treatment of long-standing kidney disease with GUTB.

## Appendices

Appendix 1

**Table 4:** Timeline of clinical presentation, diagnostic workup, treatment and outcome (case 2). USG = ultrasonography; FNAC = fine needle aspiration cytology; ZN = Ziehl Neelsen; ATT = anti-tubercular therapy; MDR-TB = multidrug-resistant TB; MGIT: Mycobacterial Growth Indicator Tube; AFB = acid-fast bacilli; HPE = histopathology; DJ = double J stent; CECT = contrast-enhanced computed tomography.

Timeline	Clinical Events / Investigations	Findings / Management
6 months before presenting to our institute	Left flank pain and testicular swelling	Progressive symptoms
2 months before presenting to our institute	Burning micturition	Lower urinary tract symptoms
February 2014 (Presentation)	Clinical evaluation	Weight loss; no past TB history
Initial workup	USG abdomen/pelvis and epididymis FNAC, urine sample was negative for microbiological analysis (GeneXpert and ZN staining)	Inconclusive findings (Negative for *Mycobacterium tuberculosis*)
Initial management	Histopathology	Granuloma; ATT started on the basis of HPE report
2 months after intensive phase of ATT	Follow-up	Persistent symptoms; DJ stenting
5 months after ATT	Clinical reassessment	Worsening symptoms; nephrostomy and biopsy
5 months after presentation	CECT and microbiological evaluation of semen sample	GeneXpert: Rifampicin-resistant *M. tuberculosis*
After MDR-TB diagnosis	MDR-TB treatment	MDR regimen initiated
6 months of MDR-TB	Follow-up	Clinical improvement; semen sample negative
